# Deep Learning Algorithms Correctly Classify *Brassica rapa* Varieties Using Digital Images

**DOI:** 10.3389/fpls.2021.738685

**Published:** 2021-09-29

**Authors:** Minah Jung, Jong Seob Song, Seongmin Hong, SunWoo Kim, Sangjin Go, Yong Pyo Lim, Juhan Park, Sung Goo Park, Yong-Min Kim

**Affiliations:** ^1^Department of Functional Genomics, KRIBB School of Biological Science, Korea University of Science and Technology, Daejeon, South Korea; ^2^Euclidsoft Co., Ltd, Daejeon, South Korea; ^3^Genome Editing Research Center, Korea Research Institute of Bioscience and Biotechnology, Daejeon, South Korea; ^4^Molecular Genetics and Genomics Laboratory, Department of Horticulture, College of Agriculture and Life Science, Chungnam National University, Daejeon, South Korea; ^5^Department of Bio-AI Convergence, Chungnam National University, Daejeon, South Korea; ^6^Department of Horticulture, Gyeongsang National University, Jinju, South Korea; ^7^Disease Target Structure Research Center, Korea Research Institute of Bioscience and Biotechnology, Daejeon, South Korea

**Keywords:** artificial intelligence, deep learning, classification model, phenotypic analysis, *Brassica rapa* (Brassicaceae)

## Abstract

Efficient and accurate methods of analysis are needed for the huge amount of biological data that have accumulated in various research fields, including genomics, phenomics, and genetics. Artificial intelligence (AI)-based analysis is one promising method to manipulate biological data. To this end, various algorithms have been developed and applied in fields such as disease diagnosis, species classification, and object prediction. In the field of phenomics, classification of accessions and variants is important for basic science and industrial applications. To construct AI-based classification models, three types of phenotypic image data were generated from 156 *Brassica rapa* core collections, and classification analyses were carried out using four different convolutional neural network architectures. The results of lateral view data showed higher accuracy compared with top view data. Furthermore, the relatively low accuracy of ResNet50 architecture suggested that definition and estimation of similarity index of phenotypic data were required before the selection of deep learning architectures.

## Introduction

One of the major features of modern science is convergent analyses using heterogeneous technologies from multiple and independent fields to analyze huge amounts of data. To manipulate these data, artificial intelligence (AI) technology has come into the spotlight. Deep learning is a type of AI that uses computer algorithms based on artificial neural networks (ANNs), which mimic the principles and structure of human neural networks to emulate human cognitive processes (Chauhan et al., 2018). In an ANN, artificial neurons (nodes) combine synapses to form a network and strengthen synapses through learning, thus acquiring problem-solving capabilities. An ANN consists of three major components: an input layer that receives data, an output layer that presents the results of analysis, and hidden layers that exist between the input and output layers. To construct an analytic model that uses deep learning, the numbers of nodes and hidden layers must be specified. Research has shown that the outcome of machine learning can be improved by increasing the number of hidden layers in the model. A machine learning method with two or more hidden layers is referred to as a deep neural network (DNN).

An ANN consisting only of a single fully connected layer, called a “fully connected neural network,” is usually used for image analysis with one-dimensional input data, which requires the dimensionality of the data to be reduced from three to one. This results in a lack of information for the AI neural network to use in extracting and learning features, resulting in limited accuracy. To overcome the limitations of the fully connected neural network, a different model called the convolutional neural network (CNN) was developed for the analysis of image or video data. A CNN consists of two layers: a convolutional layer and a pooling layer (Lecun et al., 1998). The convolutional layer is a prerequisite that reflects the activation function after applying a filter to the input data. The pooling layer is used to reduce the size of the activation map or to highlight specific data. Then, features of images were extracted through sequential analyses of two layers, namely, convolution and pooling layers. The fully connected neural network, the CNN, maintains the dimensions of the image data in each layer. For image analyses using deep learning, new CNN architectures are developed every year and presented in the ImageNet Large Scale Visual Recognition Competition. Thus, year after year, errors are reduced and accuracy is increased by changing the layer composition, depth, and calculation methods used in CNNs. In previous studies, several applications of CNN architectures showed outstanding results in the competition in the past decade (Dhaka et al., 2021) such as AlexNet (Krizhevsky et al., 2012), VGG19 (Simonyan and Zisserman, 2014), Inception v3 (Szegedy et al., 2016), Inception v4 (Szegedy et al., 2017), GoogLeNet (Szegedy et al., 2015), and ResNet50 (He et al., 2016), DenseNet121 (Huang et al., 2017), and SqueezeNet (Iandola et al., 2016).

As AI research becomes more popular, applications of AI have rapidly expanded to various research fields. In biology, AI-based analysis is used for detection, classification, and recognition with genomic and phenotypic data from humans, animals, and plants. In human research, AI-based approaches are used to classify pathogens into genetic subgroups (Prajapati et al., 2017; Sardogan et al., 2018), distinguish patient groups with different risk factors, and detect objects in images that can be used for diagnosis (Ubbens et al., 2018; Jiang et al., 2020). Animal data are also used to classify or diagnose diseases (Banzato et al., 2018a,b; Choi et al., 2018; Kim et al., 2019) and to study animal cognition (Hao et al., 2019; Yudin et al., 2019; Mohammed and Hussain, 2021). In plants, AI-based image analyses can be used to recognize specific tissues (i.e., flowers and fruits), detect diseases (Wozniak and Połap, 2018; Maeda-Gutierrez et al., 2020), and classify species, cultivars, and lineages (Lee et al., 2015; Grinblat et al., 2016; Hedjazi et al., 2017).

Plant classification plays important roles in the preservation of biodiversity, maintenance of economically important crops for food security, and discovery of new therapeutic substances, such as Tamiflu® from star anise (*Illicium verum*) and Artemisinin from sweet wormwood (*Artemisia annua*) (Ingram and Porter, 2015). The classification of plant accessions or species was traditionally carried out by grow-out tests based on phenotypes or morphologies. In recent decades, an explosion in next-generation sequencing capabilities has led to the widespread use of genetic information to classify plants. AI-based technologies now have the potential to revolutionize basic plant science, as well as breeding programs, by allowing rapid, noninvasive identification of plant varieties on the basis of digital images that can be easily acquired in high volume and at low cost.

We used four different CNN architectures to construct deep learning models to classify accessions from the *Brassica rapa* core collection on the basis of digital images. Each accession belongs to one of four groups in the core collection: Chinese accessions, early introduced accessions, Korean breeding accessions, and non-pekinensis accessions. The task of the deep learning models was to assign individual plants to the correct group using data from a single image. Four image datasets of 156 different accessions were generated. The first three datasets consisted of images taken from above the plants (top view), whereas the fourth dataset consisted of images taken from the side of the plants (lateral view). Each dataset was divided into a training set and a test set, and classification models were constructed using the AlexNet, VGG19, GoogLeNet, and ResNet50 architectures with over 50 iterations with randomly chosen data from the training sets. The results showed that the accuracy was generally higher for the lateral view images than for the top view images. Comparisons among the four architectures revealed that the GoogLeNet and VGG19 architectures had the highest accuracy with the top view images and the lateral view images, respectively, whereas the ResNet50 architecture had the lowest accuracy regardless of the dataset used.

## Methods

### Plant Materials and Generation of Plant Images

**Figure 1:**
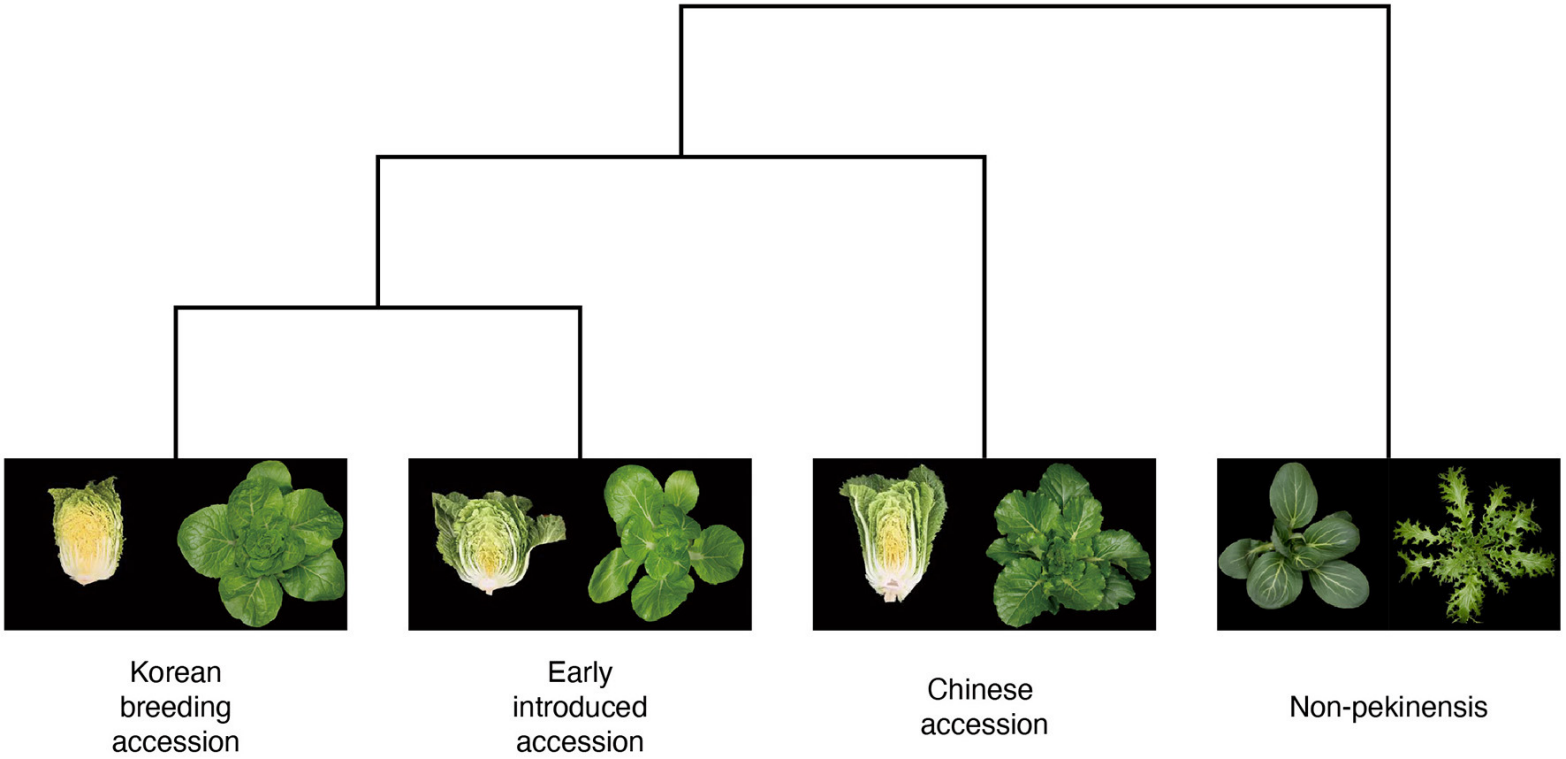
Phylogenetic tree of *Brassica rapa* with phenotypic images. The phylogenetic tree shows breeding history of four different groups (Korean breeding accession, early introduced accession, Chinese accession, and non-pekinensis) from Chinese cabbage in the east Asia (Ramchiary et al., 2011). Image data were generated from top (four groups) and lateral views (three groups) of plants from the *B. rapa* core collections. The trait of forming leafy head is the main factor to distinguish subspecies *pekinensis*.

We used 156 lines of the *B. rapa* core collection to produce three datasets for the development of classification models based on the morphology of Chinese cabbage (Pang et al., 2015). The individual lines in the core collection are classified as Chinese accessions, early introduced accessions, Korean breeding accessions, or non-pekinensis accessions depending on their geographic origin (Figure 1). The Chinese accessions include species native to China. The early introduced accessions are a group of lines that were imported to Korea in the early 1900s. The Korean breeding accessions are lines that are currently used by breeding companies in Korea. The non-pekinensis accessions comprise various subspecies of *B. rapa* including oil seed types, bok choy, turnip, and others. All accessions were cultivated in trial fields at the Chungnam National University from 2018 to 2021 to generate the top view and lateral view images.

Images of individual plants were generated with a digital single-lens reflex camera (Nikon D5300, 18-55 mm VR II). To create lateral view images of heading traits, plants were grown for 10 weeks in trial fields with 10 replicates per accession in 2018. Ten individuals per accession were then harvested, and a representative individual was selected to generate lateral view images. The images were photographed from the cross-sectional side of Chinese cabbages forming leafy heads. To generate top view images, we considered environmental conditions for core collection. The core collection contained various growing conditions such as spring-, summer-, autumn-, and winter-(southern part of Korea) harvest phenotypes. Thus, we grew the core collection in two conditions: (1) from autumn in 2020 (top views 1 and 2) and (2) from spring to summer in 2021 (top view 3). In total, five plants per accession of core collection were grown in the green house to maintain the same growth condition. Images were generated at the end of the 1st (2021), 7th (2020), and 9th (2020) week after planting using a customized photograph booth to provide the same light condition by blocking external light. Obtained phenotype data from the 7th and 9th week were grouped as top views 1 and 2, respectively. Then, phenotype data from the 1st week were grouped as top view 3. Among the 156 accessions, a total of three accessions, one Chinese and two non-pekinensis, could not be germinated and were excluded. In addition, three accessions showed an early flowering phenotype and were also excluded. In total, 2,138 images of 150 accessions were used for analysis (Table 1).

**Table 1:** Datasets used for analysis.

	**Accessions (accessions/images)**			
	**Chinese**	**Korean breeding**	**Early introduced**	**Non-pekinensis**
Top view 1	45/220	54/266	33/164	18/86
Top view 2	35/171	53/250	30/144	4/16
Top view 3	47/235	53/265	30/150	13/65
Lateral view	25/51	25/37	9/18	–/–
**Total**	47/677	54/818	33/476	18/167

### Preparation of Datasets for the Image Classification Models

**Figure 2:**
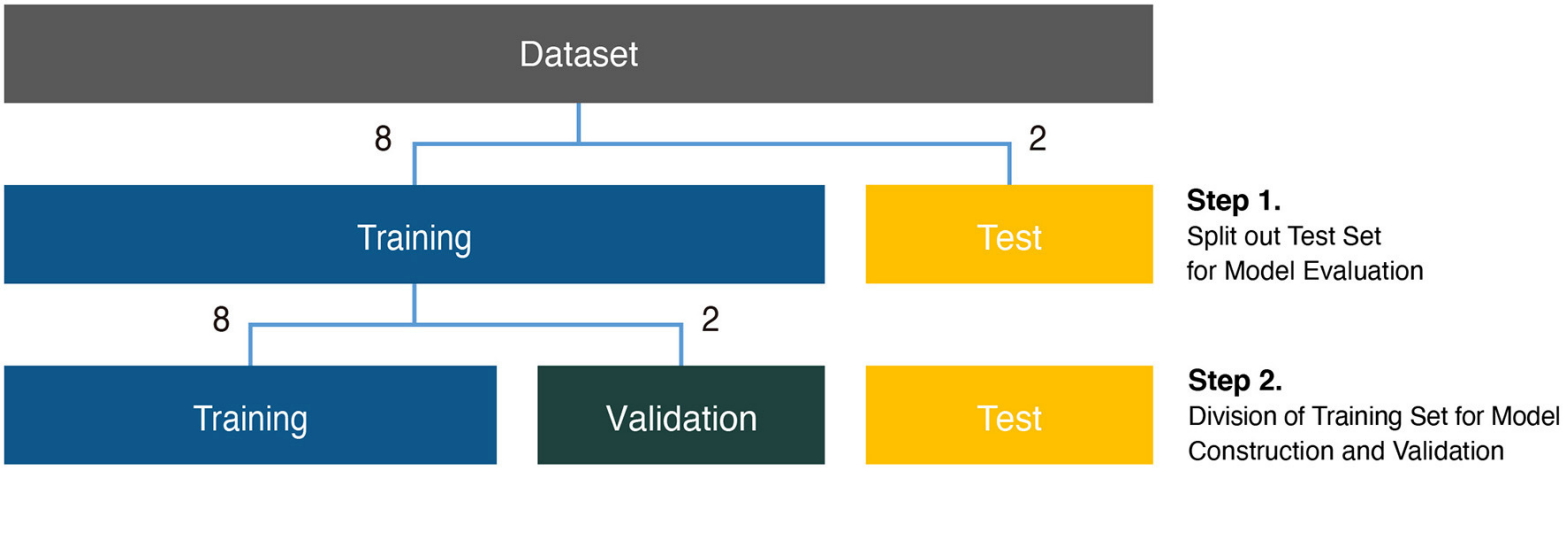
Analysis scheme for phenotypic classification. Each dataset was divided into training, validation, and test sets for construction of classification models. The size ratio of the training and test sets was 8:2 for the model evaluation (Step 1). The training set was subdivided at an 8:2 size ratio for the model construction and validation (Step 2).

Before analysis, the background of the subject in each image was erased for effective identification. Next, the images were resized to 224 pixels, which is a size commonly used in CNN analysis (Zeiler and Fergus, 2014). Then, to obtain more images for the training model, data augmentation was performed using rotation by 90°, 180°, and 270° (Shorten and Khoshgoftaar, 2019) (Supplementary Figure 1). In the case of the lateral view dataset, we had more rotation augmentation with +/- 10 degrees because of the relatively small sample size. Each dataset was divided into a training set and a test set with a sample size ratio of 8–2 between the two sets (Step 1 in Figure 2). The initial training set was further divided into a final training set and a validation set, again with a sample size ratio of 8–2 between the two sets (Step 2 in Figure 2). The final training set was used to construct the models, and the validation set was used to advance the model through hyperparameter tuning.

### Application of the Four Pretrained CNN Models

**Figure 3:**
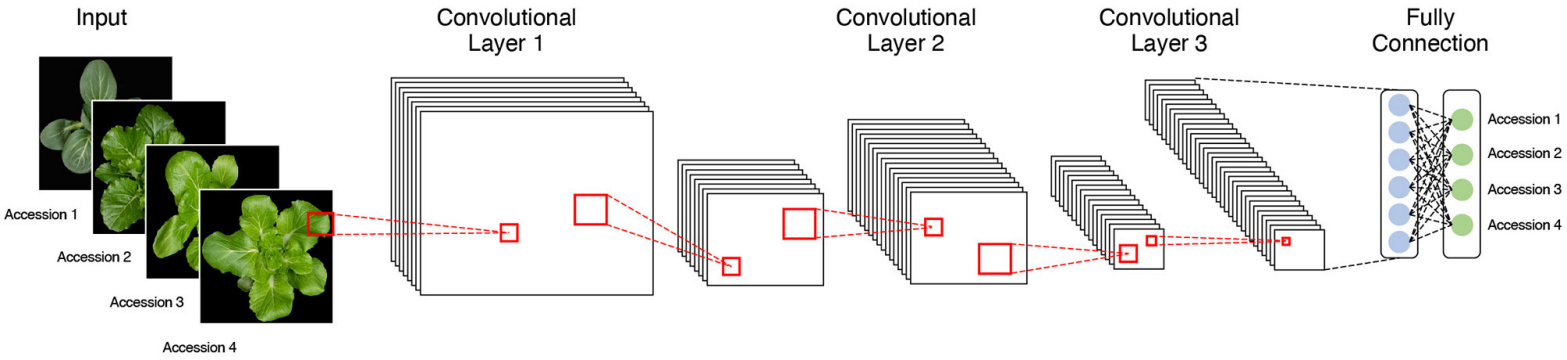
The basic structure of a convolutional neural network (CNN) analysis with image data as input. The classification model is composed of multiple convolution and pooling layers.

We used four pretrained CNN models, namely, AlexNet, VGG19, GoogLeNet, and ResNet50, to classify each accession on the basis of phenotypic image data (Figure 3). Each architecture has characteristics that distinguish it from the others. AlexNet is composed of five convolutional layers and three fully connected layers and uses a “dropout” function to avoid overfitting by switching off certain neurons. It performs parallel computation with two graphics processing units (GPUs). VGG19 consists of 19 layers and uses a large 3 × 3 kernel size filter, which can increase the depth of the network. GoogLeNet comprises 22 layers and has an “inception module” consisting of 1 × 1, 3 × 3, and 5 × 5 convolutions, which enables dimensionality reduction. ResNet50 creates an identity block with a shortcut connection that skips one or more layers on the basic multilayer structure. The analysis performances of CNN architectures, such as GoogLeNet and VGG19 with deep layers, were better than those with fewer layers like AlexNet (Table 2). However, constructing too many layers is inefficient because it takes much time and effort to calculate. Among pretrained models with more than a certain number of layers, a model with a large number of hyperparameters such as ResNet50 has good analysis performance. If precise tuning of hyperparameters was involved, the accuracy was relatively higher than that of the model with deeper layers (Dhaka et al., 2021).

**Table 2:** Features of the four pretrained models.

**Architecture**	**Year**	**# Layers (Convolutional + fully connected)**	**Model description**
LeNet-5	1998	7 (5+2)	Introduction of CNN Concepts
AlexNet	2012	8 (5+3)	Parallel computing with 2 GPUs
VGG19	2014	19 (16+3)	Multiple layers
GoogLeNet	2014	22 (21+1)	Inception module
ResNet50	2015	50 (49+1)	Skip connection
SqueezeNet	2016	Squeeze + Expand layers	Lightweight model with small size
Inception v3	2016	52 (42+10)	Improvement from GoogLeNet
Inception v4	2017	75 convolution layers	More layers with better performance rather than inception v3
DenseNet-121	2017	117 convolution + 3 transition + 1 classification layers	Connected with all layers

### Improvement of the Models Through Hyperparameter Tuning

To optimize each pretrained model for each dataset (top view or lateral view), we tuned the hyperparameters by changing the learning rate and optimizer. The batch size was determined based on the sample size of each dataset. “Softmax” was used as an activation function, which is a common practice in CNN analysis. Two optimizers were considered, “A Method for Stochastic Optimization” (ADAM) (Kingma and Adam, 2018) and “Stochastic Gradient Descent” (SGD) (Bottou, 2010). “Categorical Crossentropy,” which is known to be suitable for classification, was used as a loss function to reduce loss, or the difference between actual and predicted values. To prevent overfitting, an “earlystopping” function that stops the training of the model under certain conditions was applied using the loss of the validation set.

Each model was tested with 50 iterations using the training set with random sampling and replacement. For each architecture, the model showing the highest accuracy after validation was selected as the final classification model. Then, the final classification models were tested using the test datasets, which were not used at all in the construction of the classification model. Four pretrained models were created with python (interface) and keras (framework). In addition, all analyses were executed using a Tesla V100 GPU with 32 GB video random access memory (VRAM) and a 112 core process central processing unit (CPU).

## Results

### Construction of the Deep Learning Classification Models

We carried out CNN analysis using the four pretrained models and four different image datasets (three top view and one lateral view). To implement a model suitable for each dataset, we tuned hyperparameters such as batch size, optimizer, epoch, and learning rate by iterative validation. The batch size was tuned according to the sample size of the dataset, resulting in a batch size of 2 for the top view datasets and 17 for the lateral view datasets. SGD was chosen as the optimizer for all models and datasets except for the ResNet50 architecture with the top view 1 dataset, for which ADAM was selected. The learning rate was adjusted according to overshooting or learning time, and an appropriate rate from 0.0001 to 0.001 was set for each dataset and architecture. The epoch was set to 500 for all datasets (Table 3).

**Table 3:** Optimized hyperparameter for model advancement.

**Hyperparameter**	**Value**			
	**ResNet50**	**AlexNet**	**GoogLeNet**	**VGG19**
Batch size	Top: 2			
	Lateral: 17			
Activation	Softmax			
Optimizer	ADAM	SGD	SGD	SGD
Learning rate	Top 1: 1e−4	Top 1: 1e−5	Top 1: 1e−4	Top 1: 1e−3
	Top 2: 1e−5	Top 2: 5 × 1e−4	Top 2: 1e−4	Top 2: 5 × 1e−4
	Top 3: 1e−4	Top 3: 1e−4	Top 3: 1e−4	Top 3: 5 × 1e−3
	Lateral:1e−4	Lateral:1e−4	Lateral:5 × 1e−4	Lateral:1e−4
Epoch	500			
Early stopping	5			
Loss function	Categorical cross-entropy			

### Classification of Individual Top View Images Using the CNN Models

**Figure 4:**
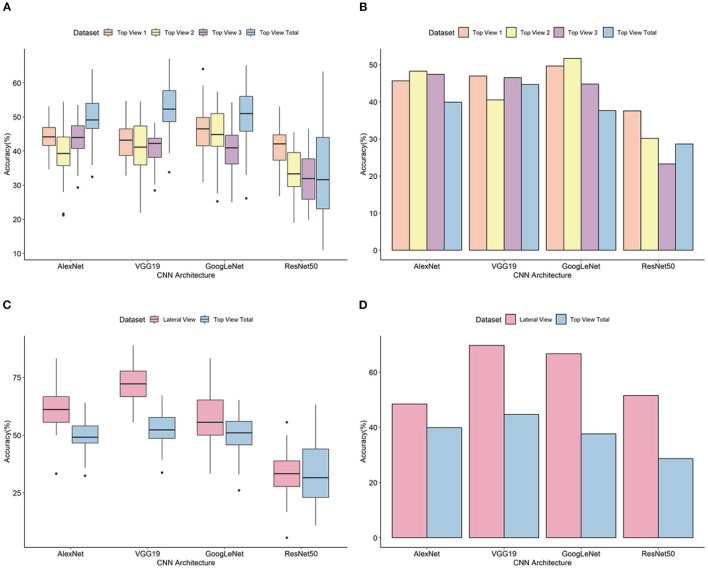
Validation and evaluation of the classification models.**(A)** Prediction accuracy of top view datasets based on 50 iterative validations. **(B)** Prediction accuracy using the test set of top view datasets. **(C)** Prediction accuracy of top and lateral view datasets based on 50 iterative validations. **(D)** Prediction accuracy using the test set of top and lateral view datasets. The horizontal axis indicates the analytical architectures, and the vertical axis indicates the prediction accuracy (%). Orange, yellow, purple, light blue, and pink colors stand for types of image dataset.

A classification model was designed for each of three independent top view datasets (top view 1, top view 2, and top view 3). For the top view 1 dataset, the average classification accuracy based on 50 iterative validations was 43.44%. The minimum accuracy was 26.77% with ResNet50, and the maximum accuracy was 64.06% with GoogLeNet (Figure 4 and Supplementary Table 1). A model showing high accuracy for each architecture was selected as the final classification model (Supplementary Figure 2). Evaluation of the final classification model using 154 images in the test set showed accuracies of 45.64% (AlexNet), 46.98% (VGG19), 49.66% (GoogLeNet), and 37.58% (ResNet50) (Figure 4). Whereas the classification for non-pekinensis accessions and Korean breeding accessions was accurate regardless of the architecture, Chinese accessions often varied depending on the architecture, and most cases of misclassification were classified as Korean breeding accession. In case of early introduced accession, the prediction performance was the lowest, and it was misclassified into Korean breeding accession or Chinese accession (Supplementary Table 2).

For the top view 2 dataset, the average prediction accuracy based on 50 iterative validations was 39.78%. The minimum accuracy was 19.00% with ResNet50, and the maximum was 57.43% with GoogLeNet (Figure 4 and Supplementary Table 3). Evaluation of the final classification model using 116 images in the test set showed accuracies of 48.28% (AlexNet), 40.52% (VGG19), 51.72% (GoogLeNet), and 30.17% (ResNet50) (Figure 4 and Supplementary Figure 3). The classifications for Chinese accessions and early introduced accessions varied among the different architectures. For the Chinese accessions, accuracy differences of more than two times were shown in accordance with architecture. According to the CNN architecture, the classification test accuracy of early introduced accession of the model was large difference until 31.03%, and most misclassified cases were classified as Korean breeding accession or Chinese accession (Supplementary Table 4).

For the top view 3 dataset, the average prediction accuracy based on 50 iterative validations was 41.06%. The minimum accuracy was 13.13% with ResNet50, and the maximum was 60.19% with GoogLeNet (Figure 4 and Supplementary Table 5). Evaluation of the final classification model using 116 images in the test set showed accuracies of 47.41% (AlexNet), 46.55% (VGG19), 44.83% (GoogLeNet), and 23.28% (ResNet50) (Figure 4 and Supplementary Figure 4). Among them, test sets from the group of Chinese accession were misclassified as the group of Korean breeding accession or group of early introduced accession. In addition, classification errors classified as the group of Chinese accession or Korean breeding were also occurred in the group of early introduced accession. Like top views 1 and 2, top view 3 dataset also showed high differences among architectures shown as top views 1 and 2 in the group of Chinese and early introduced accession while Korean breeding accessions were correctly classified regardless of architectures (Supplementary Table 6).

### Classification of Whole Top View Images Using the CNN Models

To confirm performance of classification for the top view phenotypic images regardless of developmental stages, classification analysis of the top view dataset was performed using all of the top view data including top view 1, 2, and 3. For all of the top view dataset, the average prediction accuracy based on 50 iterative validations was 37.46%. The minimum accuracy was 18.26% with ResNet50, and the maximum was 51.69% with VGG19 (Figure 4 and Supplementary Table 7). Model with highest validation accuracy, 64.00, 67.16, 65.17 and 63.29% was selected as the final classification model, each architecture respectively. Evaluation of the final classification model using 116 images in the test set showed accuracies of 39.89% (AlexNet), 44.66% (VGG19), 37.64% (GoogLeNet), and 31.74% (ResNet50) (Figure 4 and Supplementary Figure 5). As more data were acquired, it is encouraging that non-pekinensis classification accuracy increases. In individual datasets, it was difficult to classify non-pekinensis due to the small number of data available for model construction and testing, but more numbers of data gathered together and the prediction increased to non-pekinensis. Similar to individual top view datasets, the Korean breeding accession was best predicted with high accuracy, followed by Chinese accession. Early introduced accession was incorrectly classified as Korean breeding access or Chinese accession in all models except ResNet50 (Supplementary Table 8).

Furthermore, a combination of training set and test set was designed to investigate classification accuracy by developmental stages (Supplementary Table 9). For example, similar developmental stages, top views 1 and 2, were used as training set and early developmental stage, top view 3, was used as test set. Accuracy of test set in top view 1 was predicted as 44.12% (Supplementary Table 9), and top view 2 was predicted as the accuracy of 46.00% (Supplementary Table 10). However, top view 3 was predicted as the accuracy of 36.82% (Supplementary Table 11). These results suggested that phenotypes of core collection in early developmental stages were different from those of mature stages and that similar developmental stages were required to construct a classification model in early developmental stages.

### Classification of Lateral View Images Using the CNN Models

For the lateral view dataset, the deep learning analysis was carried out with ±10° rotation augmentation. In addition, no lateral view images of the non-pekinensis accessions were generated. Therefore, the deep learning analysis was carried out using images of only three groups. The classification accuracy based on the validation results ranged from 13.04 to 88.89%, with an average of 58.34% (Figure 4 and Supplementary Table 12). Evaluation of the final classification model with the test images showed classification accuracies of 48.48% (AlexNet), 69.70% (VGG19), 66.67% (GoogLeNet), and 51.52% (ResNet50) (Figure 4 and Supplementary Figure 6). Both the Korean breeding accessions and the Chinese accessions were well classified. For the Chinese accessions, 9 of the 16 test images were correctly classified with all the architectures. In the case of the early introduced accessions, three test images were classified incorrectly by all of the architectures (Supplementary Table 13).

To compare the classification accuracy of lateral and top view datasets, classification of top view 2 dataset except non-pekinensis was carried out using four architectures. Top view 2 and lateral view datasets were generated at a similar developmental stage. The average prediction accuracies of the lateral view dataset based on 50 iterative validations were 52.00, 63.27, 65.22, and 59.57% from AlexNet, VGG19, GoogLeNet, and ResNet50, respectively. The evaluation of the top view 2 (three groups) classification model showed accuracies of 46.43% (AlexNet), 55.36% (VGG19), 64.29% (GoogLeNet), and 37.50% (ResNet50). For all architectures, the accuracy of lateral view was higher than that of top view classification results (Supplementary Figure 7).

### Comparison of CNN Architecture Performances

Final classification models were constructed using the four different pretrained CNN architectures. The accuracies of classification for the test set of the top view 1 dataset were 45.64% (AlexNet), 46.98% (VGG19), 49.66% (GoogLeNet), and 51.52% (ResNet50). For the top view 2 dataset, the accuracies for the validation set were 48.28% (AlexNet), 40.52% (VGG19), 51.72% (GoogLeNet), and 30.72% (ResNet50). For the top view 3 dataset, the accuracies for the test set were 47.41% (AlexNet), 46.55% (VGG19), 44.83% (GoogLeNet), and 23.28% (ResNet50). For the lateral view dataset, the classification accuracies for the test set were 56.94% (AlexNet), 72.22% (VGG19), 61.59% (GoogLeNet), and 41.61% (ResNet50). The GoogLeNet architecture gave the most accurate classifications for top view 1 and 2 datasets, and lateral view and all top view dataset were classified with high accuracy from the VGG19 model. The highest accuracy for any architecture was 69.70% for the VGG19 architecture with the lateral view dataset. The highest accuracies for the AlexNet and GoogLeNet architectures with the lateral view dataset were 48.48 and 66.67%, respectively. The ResNet50 architecture showed the lowest accuracies of 37.58, 30.17, 23.28, and 28.65% for the top view 1, top view 2, top view 3, and all top view datasets, respectively. These results suggested that ResNet50 was not appropriate for the classification of our *B. rapa* phenotypes because of its analytical algorithm.

### Pairwise Comparisons Between Pairs of Accession Types

**Figure 5:**
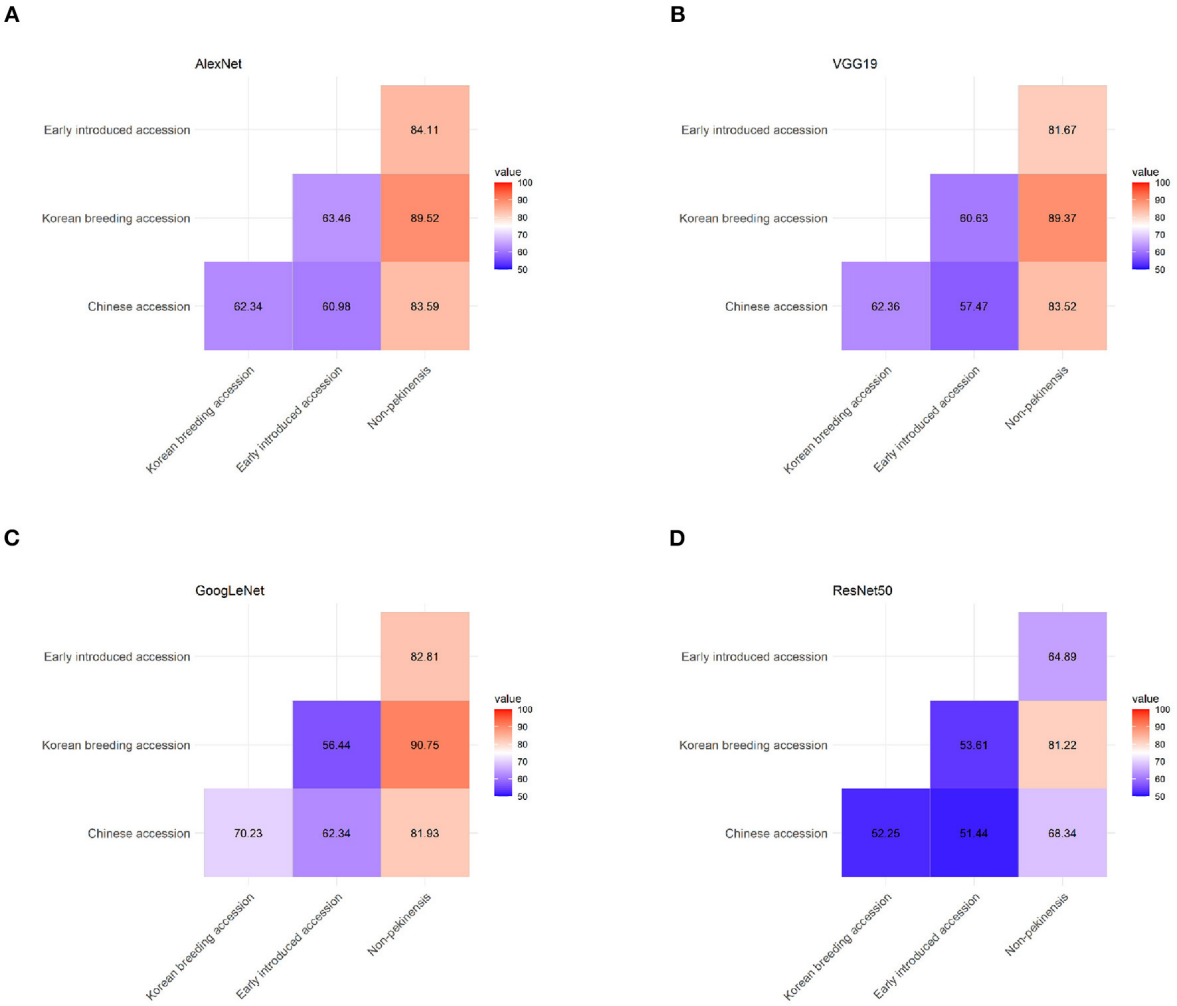
Classification accuracy in pairwise tests using the four types of accessions (Chinese, early introduced, Korean breeding, and non-pekinensis) with four different architectures: **(A)** AlexNet, **(B)** VGG19, **(C)** GoogLeNet, and **(D)** ResNet50.

We tested the ability of the final classification model to correctly classify pooled images of pairs of accession types from the top view 1 dataset with each of the four architectures (Figure 5). The non-pekinensis accessions were classified as the most accurate overall in the pairwise tests. The highest accuracy for any pair of accession types was achieved with the non-pekinensis accessions and the Korean breeding accessions, which produced an average accuracy of 87.72% over 50 iterations and a maximum accuracy of 90.75% with the GoogLeNet architecture (Figure 5C). The average accuracies achieved with the non-pekinensis accessions and the Chinese and early introduced accessions were 79.34 and 78.37%, respectively. In contrast to the non-pekinensis accessions, the early introduced accessions were relatively poorly classified overall, reaching only 58.54% accuracy in pairwise tests with the Korean breeding accessions and 61.79% accuracy in pairwise tests with the Chinese accessions. These results suggest that the early introduced accessions share some phenotypic features with the Korean breeding and Chinese accessions, which led to classification errors.

## Discussions

Huge amounts of phenotypic data are accumulating in the biological sciences. As the technology for smart farms advances, the generation and analysis of image-based phenotypic data will play a curial role in agriculture. Hence, the development of classification models using deep learning is important for both basic research and applied science. To this end, we performed deep learning-based classification analyses using images of *B. rapa*. Our results showed that deep learning architectures were able to correctly classify top view images of *B. rapa* plants more accurately at 7 weeks after planting (top view 1 dataset, average accuracy = 43.44%) than at 1 week after planting (top view 3 dataset, average accuracy = 41.06%), 9 weeks after planting (top view 2 dataset, average accuracy = 39.78%), and top view dataset of several timepoint (top view dataset, average accuracy = 37.46%). The final classification models used to classify each dataset were determined on the basis of 50 iterations with validation image sets and subsequently evaluated with independent data. The evaluation results showed that the accuracy of classification for the top view 1 dataset was 2.29% higher than that for the top view 2 dataset and 4.45% higher than that for the top view 3 dataset. These results indicated that similar developmental stages were shown better performance compared with different development stages (Supplementary Tables 9–11). In addition, more images are needed to construct classification models for various timepoint datasets. The prediction accuracies for the lateral view dataset were higher than those for the top view datasets. This is because the lateral view images showed diverse colors and more features than the top view images, such as the thickness of the leaves and the entire shape and stem of the plant.

The classification accuracies for the validation sets (step 1) and the test sets (step 2) depended on the type of architecture used (Figure 4). In the validation step, VGG19 showed the highest accuracy for the lateral view dataset, whereas GoogLeNet had the highest accuracy for the top view datasets. GoogLeNet showed the highest accuracy for all top view datasets in the test step. ResNet50 showed the lowest accuracy for all the datasets and was about 20% less accurate than GoogLeNet using the top view dataset in the test step. In previous studies, ResNet50 showed a relatively low performance to classify homogenous or highly similar images (Rudakov et al., 2018; Hassan et al., 2021). Therefore, the low accuracy of ResNet50 in our experiments might have been caused by a high degree of similarity among the images.

To investigate that hypothesis, we tested the architectures using pairs of accession types. The pairwise tests indicated that the classification of non-pekinensis together with any of the other three accession types was highly accurate, whereas that of the early introduced accessions was relatively inaccurate regardless of the other type of accession used. The classification accuracy for the Korean breeding accessions increased gradually depending on the other accession type in the pairwise test, with the early introduced accessions producing the lowest accuracy, the Chinese accessions producing higher accuracy, and the non-pekinensis accessions yielding the highest accuracy. According to the phylogenetic tree, the Korean breeding accessions are genetically close to the early introduced accessions and genetically distant from the non-pekinensis accessions (Figure 1). This suggests that phenotypic variances between the non-pekinensis accessions and the other accession types due to genetic dissimilarity led to high performance in the classification tests. The Korean breeding accessions were often classified accurately regardless of the deep learning architecture used, whereas the classifications of the Chinese accessions and early introduced accessions were inconsistent. These results further suggest that low accuracy in the classifications was caused by genetic differences between the accession types (Figure 1). Almost all of the early introduced accessions were imported from China in the early 1900s and have since been used as breeding sources. These accessions showed heterogenous phenotypes. On the other hand, the Korean breeding accessions have acquired homogenous phenotypes due to long-term breeding activities. In addition, the image depth for classification might affect the accuracy. The numbers of individual accessions used in the current analysis were not sufficient to train and evaluate deep learning models with very high accuracy for phenotypic classification. More images of various developmental stages would improve the accuracy of the models. Furthermore, tissue-specific and trait-specific images can be used in the future to identify trait-associated genes by correlation with genotypic data.

For applications of deep learning classification models in industrial fields, top view images are more suitable than lateral view images, although we achieved higher accuracy using the lateral view. Classification models like the ones presented in this study can be applied for nondestructive inspection of accessions and cultivars; however, improved models need to be developed, which can identify different types of plants with high accuracy using top view images.

## Data Availability Statement

The datasets presented in this study can be found in online repositories. The names of the repository/repositories and accession number(s) can be found in the article/Supplementary Material.

## Author Contributions

Y-MK conceived the project, designed the experiments, and organized the manuscript. MJ, JS, and SP performed the deep learning analysis. SH, SG, SK, and YL generated the phenotypic data. MJ and Y-MK wrote the manuscript. All authors contributed to the article and approved the submitted version.

## Funding

This work was supported by the Korea Forest Service of the Korean government through its R&D Program for Forestry Technology (Project No. 2014071H10-2122-AA04), and by the Korea Institute of Planning and Evaluation for Technology in Food, Agriculture, and Forestry through the Golden Seed Project (213006-05-5-SBG30) and the Technology Commercialization Support Program (821026-03), which is funded by a grant from the Ministry of Agriculture, Food, and Rural Affairs, the Ministry of Oceans and Fisheries, the Rural Development Administration, and the Korea Forest Service to Y-MK.

## Conflict of Interest

Author MJ, JS, and JP are employed by Euclidsoft Co., Ltd. The remaining authors declare that the research was conducted in the absence of any commercial or financial relationships that could be construed as a potential conflict of interest.

## Publisher's Note

All claims expressed in this article are solely those of the authors and do not necessarily represent those of their affiliated organizations, or those of the publisher, the editors and the reviewers. Any product that may be evaluated in this article, or claim that may be made by its manufacturer, is not guaranteed or endorsed by the publisher.

## Supplementary Material

The Supplementary Material for this article can be found online in the Dryad repository via the DOIs indicated below.

Supplementary Figure 1 Available via doi: 10.6084/m9.figshare.16620523.

Supplementary Figure 2 Available via doi: 10.6084/m9.figshare.16620538.

Supplementary Figure 3 Available via doi: 10.6084/m9.figshare.16620568.

Supplementary Figure 4 Available via doi: 10.6084/m9.figshare.16620571.

Supplementary Figure 5 Available via doi: 10.6084/m9.figshare.16620580.

Supplementary Figure 6 Available via doi: 10.6084/m9.figshare.16620592.

Supplementary Figure 7 Available via doi: 10.6084/m9.figshare.16620598.

Supplementary Tables 1–13 Available via doi: 10.6084/m9.figshare.16620604.
